# Nosema Disease of European Honey Bees

**DOI:** 10.3390/jof7090714

**Published:** 2021-08-30

**Authors:** Richard Galajda, Alexandra Valenčáková, Monika Sučik, Petra Kandráčová

**Affiliations:** Department of Biology and Physiology, University of Veterinary Medicine and Pharmacy, Komenského 73, 041 81 Košice, Slovakia; richard.galajda@uvlf.sk (R.G.); monikasucik@gmail.com (M.S.); petra.kandracova@uvlf.sk (P.K.)

**Keywords:** *Nosema apis*, *Nosema ceranae*, *Apis mellifera*

## Abstract

Nosematosis is currently a frequently discussed honey bee disease caused by two types of Microsporidia: *Nosema apis* and *Nosema ceranae*. Nosematosis as an intestinal disease caused by these species is one of the main factors associated with the weakening and loss of hives, with none of the stressors acting in isolation and all having an important synergistic or additive effect on the occurrence of parasitic infection. The most important factors are exposure to pesticides and nutritional stress, both worsening the immune response. Honey bees *Apis mellifera* become more susceptible to parasites and subsequently the disease manifests itself. Choosing the right laboratory diagnostics is important to determine the prevalence of both species. Our review summarizes the most commonly used methodologies, especially polymerase chain reaction (PCR), which is a reliable method for detecting nosematosis, as well as for distinguishing between the two species causing the disease.

## 1. Introduction

Honey bees are essential for the conservation of biodiversity and their crop pollinator services cannot be underestimated [1]. Over the last 10 years, global bee populations have been severely reduced by various environmental stressors, including, but not limited to: malnutrition, loss of food habitats, infectious exposure to viruses and parasites and exposure to pesticides and other persistent chemicals [2,3]. Each of these individual stressors represents environmental exposures that adversely affect bee health at the colony level. In addition, these environmental exposures can interact and synergistically affect the overall health of the hive. Such impaired hive health is the gateway for opportunistic pathogens. Among the most documented opportunistic infections in bees we include nosematosis, which is caused by microsporidia of the genus *Nosema* [4].

Microsporidia are a group of small spore-forming obligate intracellular eukaryotic parasites that infect a wide range of hosts from invertebrates to vertebrates, including humans and insects [5]. To date, three types of microsporidia associated with honey bee infection have been described, all three of which belong to the genus *Nosema*. Infections are known as nosematosis, whether caused by *Nosema apis* [6], *Nosema ceranae* [7] or *Nosema neumanni* [8]. The first two species have spread throughout the world and are responsible for the known symptoms of diarrhea and the reduction of bees in the hive. [9,10,11,12]. However, *N. neumanni* has recently been detected only in Ugandan bees and its implications for host bees have not yet been described in detail. *N. apis* and *N. ceranae* are generally considered to be the original parasites of, respectively, European honey bees (*A. meliffera)* and Asian honey bees (*A. ceranae)* [13]. However, *N. ceranea* has been shown to be able to effectively infect *Apis mellifera* individuals, allowing it to spread far beyond the Asian continent [9]. Natural infections in which hosts are infected with a single parasite are rare [14]. In the honey bee host, interactions between parasites can affect disease severity, and mixed *N. apis* and *N. ceranae* infections are common [15,16,17,18]. This results in a reduced colony consisting mostly of nurse bees with their queen, a state very similar to that seen in Colony Collapse Disorder (CCD). Recent survey of microflora in this reduced colonies-affected and healthy colonies (without symptoms) indicated a higher prevalence of *N. ceranae* compared to *N. apis* [19].

## 2. Biology and Pathogenesis of *Nosema* spp.

*Nosema* spp. are spore-forming microsporidia. Spores are the infectious stage and are the only stage that is able to exist outside the cell. *N. apis* spores are oval in shape, approximately 6 × 3 μm in size [20]. According to the description of Fries et al., [7], spores of *N. ceranae* are smaller and their size is around 4.7 × 2.7 μm.

**Figure 1:**
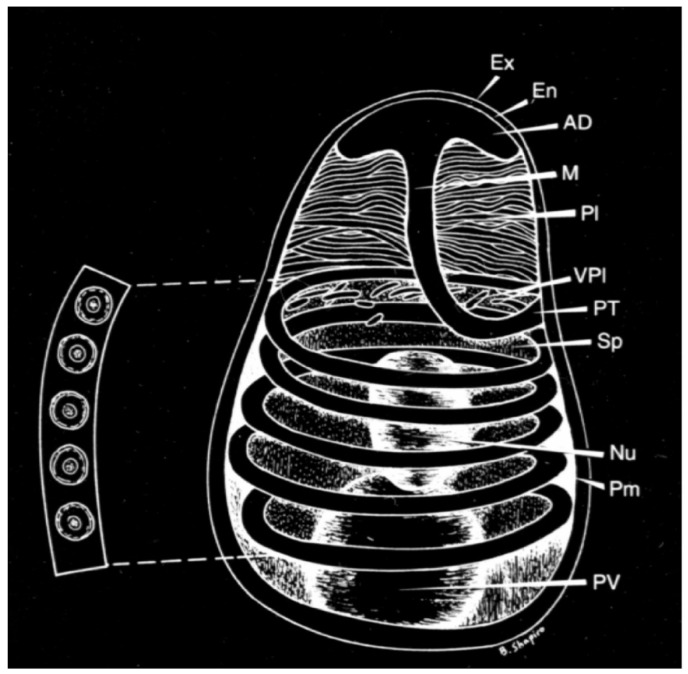
Structure of a spore of Microsporidia. Ex—outer wall of microsporidia spores, En—inner wall of microsporidia spores, AD—top of the spore, M—manubrium, Pl—lamellar polaroplast, VPl—vesicular polaroplast, PT—polar tube, Sp—sporoplasm, Nu—nucleus, Pm—arranged membranes, PV—posterior vacuole [22].

The spore in microsporidia (Figure 1) is bounded by a common membrane and two solid extracellular walls. The outer wall is formed by a dense granular protein matrix [21]. The inner wall of microsporidia spores (En) consists of alpha chitin and proteins and is at any point of the same thickness, except the apex (AD) and manubria (M), where the inner wall is thinner than in other parts. Below the membrane is the sporoplasm (Sp), which is an infectious material of microsporidia. Sporoplasm contains, depending on the type of microsporidia, one or two nuclei (Nu) in the form of diplocaryon (*Nosema* spp.,-two tightly compressed nuclei), ribosomes and other spore structures, such as: polar tube (PT) and posterior vacuole (PV; 1) which are necessary for the process of infection of the host cell The polaroplast consists of arranged membranes (Pm) located in front of the spore. The front part of the polaroplast is formed by tightly stacked membranes called lamellar polaroplast (Pl). The posterior part of the polaroplast is arranged looser than the anterior part and is called the vesicular polaroplast (VPl). The most obvious organelle associated with infection is the polar tube [22].

The polar tube is attached to the apex of the spore by means of an umbrella-like structure, also called an “anchoring disk”, from which it extends to the back of the spore. One-third to one-half of the tube is straight, the rest is twisting in a spiral around the contents of the sporoplasm. The number and arrangement of spirals and the angle of inclination of the spirals is a characteristic distinguishing feature for different species of microsporidia [23]. *N. apis* spores have a number of polar tube turns greater than 30, whereas, *N. ceranae* has a number of polar tube turns 18–21 [7]. The polar tube is terminated by a third larger organelle, the posterior vacuole. The whole germination process is usually performed in the same way in *Nosema apis* and *Nosema ceranae* in less than two seconds and the end of the emptied tube is moved at a speed of 100 μm·s^−1^ [24]. The polar tube is a very important protrusion. If there is a host cell in the vicinity, the fired tube can hit it and disrupt its membrane as a “projectile” hit target. The polar tube is completely emptied and constant pressure inside the spore (probably caused by an increase in the posterior vacuole) conducts the sporoplasm through the polar tube into the interior of the host cell. In *N. ceranae*, 16 h after entering the host cell, the sporoplasm from the tube enters the cytoplasm of the host, thereby infecting it without identifying the parasite as a foreign intruder [25].

### Life Cycle for N. ceranae

**Figure 2:**
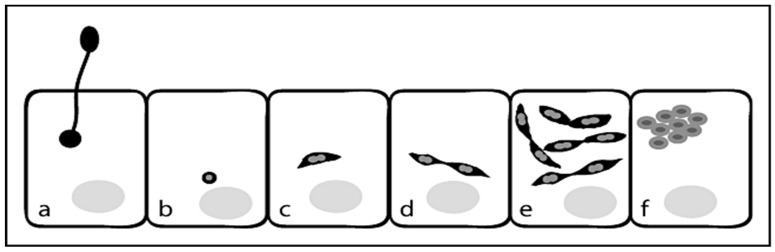
Pathogenesis and transmission of *N. ceranae* into a host cell. Schematic representation of the early events in the life cycle of *N. ceranae*: (**a**) injection of the sporoplasm into the host cell, (**b**) the sporoplasm in the host cell, (**c**) aspindle-shaped meront, (**d**) paired meronts, (**e**) several rounds of cell division, (**f**) round to oval sporonts.

The beginning of sporogony is characterized by the appearance of sporonts and later sporoblasts with round to oval morphology, a thickened plasma membrane and densely stained polaroplasts. These sporoblasts then develop into round to oval primary spores or into oval environmental spores. Spores extrude the polar tube which pierces the cell membrane of the target cell followed by injection of the sporoplasm into the host cell. The sporoplasm appears as small spherical body in the host cell and then develops into a spindle-shaped meront, which begins to divide giving rise to paired meronts. These pairs of meronts then undergo several rounds of cell division until they separate and develop into round to oval sporonts, which are condensed and characterized by a thickened plasma membrane (Figure 2) [25].

Thus, the vegetation cycle is completed after approximately 96 h and the infected cells contain a number of primary and environmental spores (H, P) [24].

In *N. apis* there are several differences, especially in the shape of meronts, which are characterized by a round to oval shape that do not form pairs as in *N. ceranae* [6,24].

## 3. Transmission

Infection occurs by the ingestion of spores in the feed, via trophallaxis [26] or perhaps after grooming of the body hairs [6]. Oral transmission via a spore-containing diet has so far been considered the most important mode of transmission of nosematosis *(Nosema apis*) in hives. During the first 5 days after the invasion of infectious spores due to the disruption of the intestinal barrier, saprophytic bacteria pass from the digestive tract into the bee hemolymph and subsequently die of septicemia. Infected intestinal epithelial cells lose plasma and later develop a peritrophic membrane, leading to impaired gastric function and imperfect digestion and trophallaxis [27].

Significantly sweeter taste of feces in nosematosis and the hygienic behavior of honey bees are reasons for ingestion, meanwhile infecting another bee. Infected honey bees in the outdoor environment contaminate food sources (water and pollen). This spreads the infection, which is also helped by the flying of bees. Bad beekeeping practice and contaminated wax foundation are other sources of spores for *Nosema* spp., as well as robbing of heavily infected hives by bees from other hives, especially in late summer. The beekeeper himself can also be a source of infection, for example by changing the queen for an infected one, or by joining or replenishing hives [28].

## 4. Pathogeny in *N. apis* and *N. ceranae*

Honey bee nosemosis is caused by representatives of two types of microsporidia, *Nosema* *ceranae* and *Nosema apis*. *N.* *ceranae* occurs stably in hives throughout the year, *N. apis* has seasonal character with a typical peak of occurrence and impact on bee colonies in winter. Whereas *N.* *ceranae* usually has an asymptomatic course, *N. apis* in its typical form causes dysentery and its presence is visibly manifested by burnt frames, combs and the outer walls of the hive [28].

Honey bees infected with *N. apis* do not show any signs of infection at first, but their lifespan is noticeably shorter as well as they have imperfectly developed vacuolar hypopharyngeal glands [29]. The infection can take on the dimensions of an epidemic and cause a significant weakening of the entire honey bee colony. Queens infected with *N. apis* often stop egg laying, especially in the spring, and die a few weeks after the onset of the infection, mostly outside the hive area. Usually, they are infected during feeding by infected workers [29].

Another effect that is observed in the presence of the parasite is the inability of honey bee to fully utilize food proteins. Their deficiency causes atrophy of the pharyngeal glands, also, in workers the inability to feed the larvae and queen, which results in premature aging of the population [30]. In acute infection, we observe the vibrations of the workers’ bodies and the enlarged abdomen [31]. Infected honey bees are affected by dysentery, which enormously promotes the spread of the disease throughout the colony (under standard conditions, bees defecate during flight outside the hive). Dysentery can be identified by dead honey bees and fecal markings around the hive [29]. The cause of dysentery is imperfect absorption of carbohydrates, due to the presence of parasite, which, especially in the winter, load the fecal sac and thus cause dysentery. Heavily nosematic honey bee colonies are likely to die in the winter [30].

In the standard course of infection, colonies are able to recover if they are infected at the beginning of the year, while those infected in the autumn often die during the hibernation period [29]. The fact is that they are losing a large part of the population and one of the reasons is the small supply of honey created during the presence of the parasite, with an estimated reduction of 20–30% [32]. It is also likely that some of the symptoms observed are the result of viruses, which often co-occur with parasites and whose effects are synergistic with each other [29].

*N. apis* infection is often associated with 3 unrelated viral infections, namely: black queen cell virus (BQCV), bee virus Y (YV) and filamentous virus (FV) [31]. In particular, the combination of *N. apis* infection with BQCV is significantly more harmful than the individual infections alone. The virus is able to add the subspecies apis virulence. It was also found to be frequent in the company of the protozoan *Malpighamoeba mellificae*, which may be the cause of the aforementioned dysentery [29].

In the case of *Nosema ceranae*, the colonies initially show no signs of infection, except for increased mortality in winter. The OIE Terrestrial Manual, Nosemosis of Honey Bees (2013), states that when an infection caused by *N*. spp. breaks out, the intestine of the honey bee changes from its original brown to white color and becomes very fragile. In the acute course of infection caused by *N*. spp. we can also notice feces on combs, hives, a strong fall of dead bees and a rapid weakening of the honey bee colony [33].

The main consequences described in infected bees include stress, “behavioral fever” as a form of social immunity, hormonal disorders, depletion of immunity and consequent reduction in life expectancy [27]. *Nosema* in honey bee fat cells causes gene expression in metabolic and nutritional pathways, which is likely to lead to transcriptional and physiological changes associated with behavioral change, decreased immune response and shortened lifespan [34]. Colonies with a chronic course of nosemosis are weak and bees are gradually declining, flying poorly and have a shortened life [35].

## 5. Prevalence of *N. apis* and *N. ceranae*

The incidence of *N. apis* correlates with the observed winter mortality, but the main problem is that the disease often occurs without losses in infected colonies [31]. In temperate climates, *N. apis* infection has a lower prevalence in summer, a small peak in autumn and a slow rise of infection during winter [6].

In the spring, when larval rearing begins, the infection increases rapidly because larval rearing begins while flying is limited during this period [29].

On the contrary, another study shows the absence of a temperature gradient, when the prevalence in colder climates did not appear lower, reaching very similar values [29]. It can be concluded that the prevalence of the parasite is variable under different conditions and colonies; e.g., Varis et al. (1992) [36] screened samples of honey bees in the United Kingdom and Finland, where they found *Nosema apis* infection in more than 1/3 of honey bee colonies showing the presence of spores.

Gisder et al. (2017) [37] presented 12-year long-term cohort study on the occurrence of *N. apis* and *N. ceranae* in northeastern Germany. Between 2005 and 2016, a cohort was sampled twice a year (spring and autumn) with approximately 230 bee colonies from 23 apiaries, resulting in a total of 5600 samples of bees subjected to microscopic and molecular analysis to determine the presence of *N. apis* infections or/and *N. ceranae*. Throughout the study period, it was possible to diagnose both *N. apis* and *N. ceranae* infections in the cohort. Logistic regression analysis of prevalence data showed a significant increase in *N. ceranae* infection over the last 12 years, both in autumn (reflecting developments in summer) and in spring (reflecting developments in winter). Cell culture experiments confirmed that *N. ceranae* has a higher proliferative potential than *N. apis* at 27 °C and 33 °C, potentially explaining the increased prevalence of *N. ceranae* in summer. In autumn, which is characterized by a generally low prevalence of infection, this increase was accompanied by a significant decrease in the prevalence of *N. apis* infection. Conversely, in the spring, in the season with a higher prevalence of infection, no significant decrease in *N. apis* infections despite a significant increase in the incidence of *N. ceranae* infections could be observed. Therefore, their data do not support the general advantage of *N. ceranae* over *N. apis* and the total replacement of *N. apis* for *N. ceranae* in the honey bee population studied [37].

In this species, the problem is that there is a lack of symptoms of infection, since dead honey bees are only rarely seen, and this could also be due to other diseases and pesticides [33]. Severe symptoms have been reported mainly in the south, rarely in temperate climates. However, it is possible that the course of infection also varies with different bee subspecies, food supply, agricultural practices in the locality, hive management or other abiotic and biotic factors [31].

Milbrath et al. (2014), [18] experimentally demonstrated higher mortality rates with mixed infection which may be due to higher spore reproduction-mixed infections resulted in a higher number of total spores, although the mechanism of this increased spore reproduction is unknown. Alternatively, these two species of Nosema can attack different molecular or physiological systems of the honey bee resulting in a synergistic effect of infections of mixed species.

Worldwide, the huge incidence of *N. ceranae* has been recorded mainly in the USA. The reason why it has become the dominant parasite in the USA remains controversial [17].

In the Czech Republic, according to a study by Kamler et al. (2011) [38] were examined 4010 hives from all regions from 113 beekeepers (Table 1). Overall incidence and prevalence of *Nosema* spp. is as follows:

In addition, a synergistic effect between the disease and the agricultural pesticides used is currently beginning to manifest itself, as the parasite increases susceptibility to chemical stressors. The results clearly show that even in the conditions of the Czech Republic, *N. ceranae* gradually becomes a dominant parasite and its progress needs to be monitored [38].

This shift in favor of *N. ceranae* is also confirmed by the observations of Staroň et al. (2012) [39], who in 2009 and 2010 monitored the incidence of both species in the Slovak Republic and confirmed the increase in the prevalence of *N. ceranae* and the decrease in *N. apis*.

The team of Botias et al., (2012) [13] conducted a survey of adult bees carried out in three Spanish regions in spring 2002 and 2007: Catalonia, Valencia (both in the Mediterranean) and Andalusia (southern Spain). Prevalence of *Nosema* spp. represented 30.2% in 2002 and 47.2% in 2007. The trend of increased prevalence was observed in all three areas and was related to an increase in the presence of *N. ceranae*. In Hungary, nationwide sampling of live adult honey bees (*Apis mellifera carnica P*.) was organized in 2010. The study involved 44 apiaries from the main geographical regions of the country. Percentage of hives infected with *Nosema* spp. ranged between 95% and 98%. The distribution of *N. ceranae* and *N. apis* infection rates during the season was homogeneous. The prevalence of *N. ceranae* was always significantly higher than that of *N. apis* [40].

Studies in Bulgarian, Belgian, Italy also have found that *N. apis* is being replaced by *N. ceranae* [18,41,42,43,44].

## 6. Laboratory Diagnosis of Nosematosis

**Figure 3:**
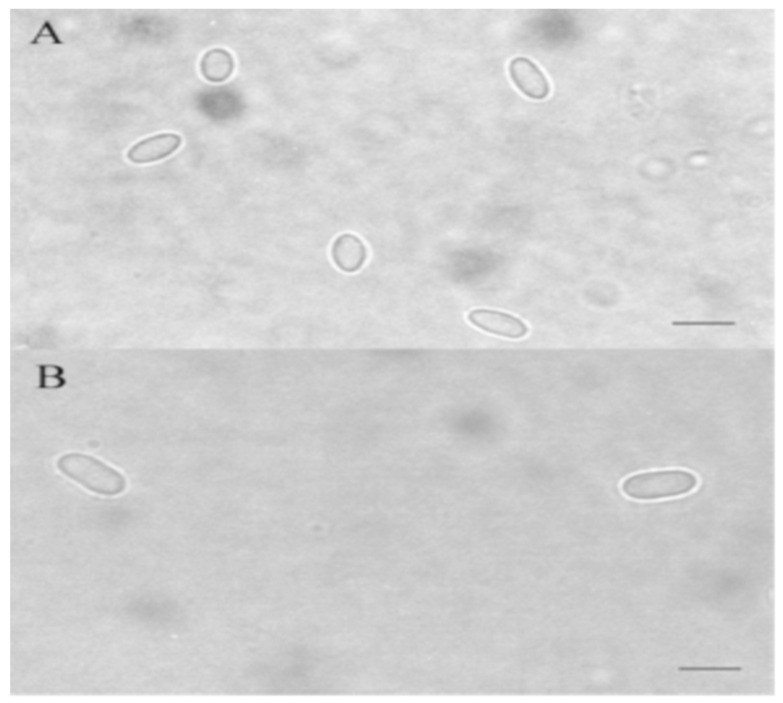
*Nosema ceranae* (**A**) and *Nosema apis* (**B**) spores in a phase contrast microscope. Sample-aqueous coating [9]. Scale bar is 5 µm.

**Figure 4:**
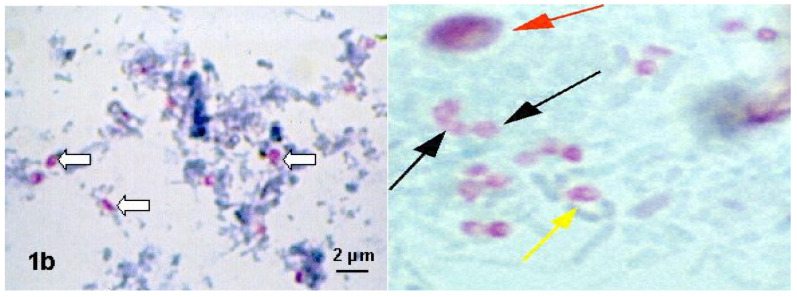
Modified chromotropic staining methods: Weber-Green and Ryan-Blue. Spores (arrows) are pinkish-red, with a light interior of the spore, or possibly with horizontal or diagonal stripes [51].

**Figure 5:**
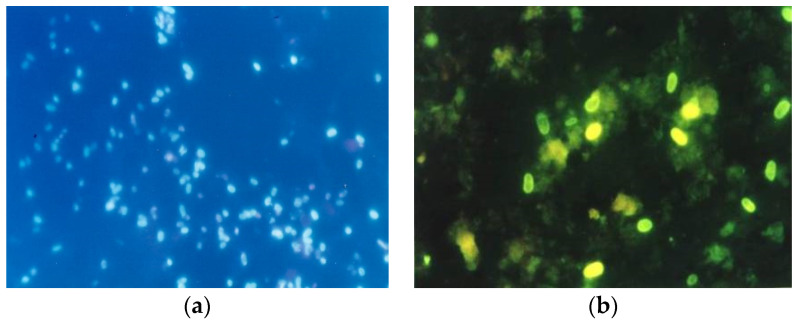
Microsporidia spores: (**a**): sample stained with calcofluoride—blue fluorescence (**b**): sample on the right stained with Rylux—green fluorescence.

When processing samples, the first step is to concentrate the microsporidia for further analysis. Ether extraction is used to concentrate and subsequently purify spores from honey bee feces by a modified sedimentation procedure in aqueous ether [45]. Sampling and sample preparation for PCR analysis consisting of homogenization, filtration and DNA extraction is described in the OIE Terrestrial Manual, Chapter 3.02.04 Nosemosis 2018, [46]. The sediment can be used for culture, for DNA isolation or after fixation on a slide with a phase contrast microscope (Figure 3) or for fluorescence staining (Figure 5) or Weber trichrome staining (Figure 4) [47,48].

There are used specific microscopic methods in the diagnosis of *Nosema* spp., specifically transmission electron microscopy and molecular analysis to clearly distinguish these species, where several PCR protocols have been described, including PCR with specific primers [49], PCR-RFLP [9,16], real-time PCR [12,19] or multiplex PCR [50].

### 6.1. Microscopic Methods in the Diagnosis of Nosema spp.

Giemsa staining may be useful in visualizing infection in tissue samples. Another method for identifying *Nosema* spp. by means of light microscopy is to stain the sample by contrast staining using toluidine blue. These staining methods reliably distinguish microsporidia from other species of microorganisms [25].

One possibility is to use modified trichrome staining. Coatings are prepared using 10 to 20 µL of a concentrated stool sample, which is thinly spread on a slide. Modified trichrome staining methods are based on the fact that penetration of the dye into the microsporidium spore is very difficult, therefore the dye content in the chromotropic 2R component is higher than that used in the Wheatley modification of Gomorrah trichrome staining [47,51].

Some of these staining methods, as for example Weber-Green modified trichrome staining (Figure 4) and Ryan-Blue modified trichrome staining are commercially available. The use of a positive control is recommended. Spore detection requires adequate illumination and magnification (oil immersion, total magnification ×1000).

The spore wall should be pinkish-red, with a light interior of the spore, or possibly with horizontal or diagonal stripes, which represents the polar tube. The background appears green or blue, depending on the method used. Bacteria, several yeast cells and some impurities (stained in various shades of pink and red) can also be stained, but the shapes and sizes of these artifacts can be helpful in distinguishing them from spores.

Specialized workplaces, which are dealing with the study of several species of microsporidia, use specific staining methods based on optical brighteners. Chemofluorescent (optical brightening) agents are complex organic chemicals containing aromatic rings in molecules, which have two basic properties. In shortwave light, completely excited, they emit light in the visible spectrum and bind more or less specifically certain substrates [48]. With the help of optical brighteners, the spores are visualized in a fluorescent microscope due to the binding of the optical brighteners to the chitin in the spore wall. Depending on the reagent used, as well as the wavelength, the walls of the microsporidial spore will fluoresce. Using Calcofluor White M2R (Sigma) and a wavelength of 395–415 (light on observation, 455 nm), the spores will appear as bluish-white or turquoise oval formations (blue fluorescence; Figure 5).

It is important to remember that this type of staining is non-specific, small fungi and some artefacts present in the stool may also fluoresce. However, if this approach is applied to samples other than feces samples, it is much easier to detect and identify microsporidial spores. Other optical brighteners that can be used include Fungi-Flour and Cellufluor (Sigma-Aldrich, St. Louis, MO, USA), Fungiqual A (Medical Diagnostics, Kandern, Germany) and Uvitex 2B, Rylux (not commercially available, but are available from the manufacturer of washing powders Ostacolor, (Pardubice, Czech Republic), which give green fluorescence when using excitation filters of 405–490 nm, color ray at 510 nm and barrier filter at 550 nm (Figure 5). This means that the spores stained with these optical brighteners will appear as green oval formations.

Transmission electron microscopy is based on the observation of microsporidia in excrement samples or bee intestine samples. Microsporidia can be identified down to the level of genus or even species, based on morphological properties. Problems with TEM include the need for a costly instrument and a laborious method for sample preparation and investigation [52]. Several sample fixation procedures are available for microsporidia studies. The number of turns of the polar tube is one of the tools that helps to distinguish the species of *Nosema* spp. [53]. Immature spores, where the tube is still evolving, can be distinguished from mature spores as well.

### 6.2. Molecular Detection of Nosema spp. (N. apis, N. ceranae and N. bombi)

In addition to the microscopic techniques described above, molecular methods have been developed for detection and identification. Molecular methods are more sensitive and species specific [11]. Molecular detection of microsporidia *Nosema* spp. in the honey bees can be used even if there is a very little of pathogen present in the sample and the pathogen has little or no effect on its host. Another advantage of molecular methods is that their extreme sensitivity of detection can provide knowledge of hitherto unknown pathways of pathogen transmission. Molecular techniques developed for the detection of *Nosema* spp. in honey bees and bumblebees (*N. apis*, *N. ceranae*, *N. bombi*), are usually based on the use of PCR (uniplex or multiplex) PCR, PCR –RFLP, qPCR [54] using a wide range of species-specific primers for PCR. Several primers for these *Nosema* species are listed in Table 2. Adequate laboratory equipment and quality know-how are a prerequisite for the use of these methods in the diagnosis of microsporidiosis.

Tests of the specificity and detection limits of nine of these primers suggest that some of them have low specificity or low levels of sensitivity [57]. In addition, the use of different molecular methods or conditions in laboratories can lead to inconsistencies.

In addition to these specific primers for conventional PCR and real-time PCR [31], protocols for the detect of *N. ceranae* and *N. apis* have been proposed (Table 1), including primers that detect both species in a single reaction [12].

In addition, PCR method has also been developed that is able to simultaneously detect three *Nosema* species with a high prevalence in European honey bee populations (*N. apis*, *N. ceranae*) using genomic DNA. DNA extraction can be performed from specific tissue (e.g., *ventriculus*, *rectum*, adipose tissue), from part of bees (e.g., *metasome*) or whole honey bees or homogenates from pooled samples [57]. PCR method to detect Nosema species amplifies a 16S rRNA (=SSU rRNA) gene fragment, which was designed based on alignment of all available sequences and data from the GenBank of 16S rRNA gene from *N. apis*, *N. bombi* and *N. ceranae*. Molecular phylogeny revealed the genetic divergence in *N. apis* and *N. ceranae* isolated from host species that were collected in different geographic regions and at different times. Phylogenetic trees *N. apis* and *N. ceranae* are based on partial sequences of SSUrRNA [62]. Each laboratory may need to optimize *de novo* primers, protocols and PCR conditions.

Visualization in multiplex PCR method is performed electrophoretically on a 1–2% agarose gel with a marker of appropriate size and then visualized by GEL RED staining and UV transilluminator photography [63] *Nosema* species can also be detected by qPCR with probes, which increases the specificity of the method [54].

## 7. Diagnostic Procedures Used at Our Workplace

The first option—as a screening, we recommend the first use of the examination of feces scraped from the areas of the affected hives. The feces must be extracted with ether before staining. Dilute 1 g of feces in 6 mL of water, filter over the gauze, add 6 mL of diethyl ether, mix thoroughly and centrifuge for 2 min at 600× *g* [45]. The obtained sediment can be used for cultivation, for DNA isolation or, after fixation, applied to a staining slide. In our laboratory, staining with optical brighteners (RyluxD, Calcofluor, Pardubice, Czech Republic) has proved successful, with the help of which we can successfully exclude negative samples. It is very advantageous to use a positive control (e.g., deposited spores of *Nosema* spp. from older positive samples) and compare the size and shade of the fluorescence.

The second option—in the case of dead bees in affected hives or in chronic infection caused by *Nosema ceranae* (when the infection is asymptomatic) and excretion of spores by excrement may not be the rule, is another option for processing bee abdomen samples using the method described in the OIE Terestrial manual (2018).

The microscopic examination is then supplemented by DNA molecular analysis, in order to identify the type of agent. The sample must be pre-isolated before DNA isolation. At our workplace, we use a Precellys 24 sample homogenizer (Bertin Technologies, GmbH, Frankfurt am Main, Germany), with which the samples are mechanically broken with glass (0.5 mm) and zirconium beads (1.0 mm) and centrifuged at 6500 rpm/min. for 90 s in 300 µL lysis solution. Then we isolate the DNA using commercial DNA extraction kits (QIAGEN or AmpliSense, Moscow, Russia). The choice of method used to isolate DNA can significantly affect the sensitivity of the reaction. In our practice, it has proven to be an effective method for the molecular detection of samples infected with *Nosema* spp. which involves the use of a multiplex PCR reaction using four primers 218 MITOC F/R and 321 APIS F/R to amplify 16S rRNA gene regions (*Nosema apis* and *Nosema ceranae*), at product sizes 218–219 bp and N. apis-321 bp.

## 8. Cultivation on Cell Structures

Some microsporidia, such as species of the genera *Encephalitozoon* and *Brachiola*, although present in small numbers, have the potential to reproduce in cell cultures, making it easier to identify and analyze them at a later time. According to the available literature for microsporidia of *Nosema* spp. infecting honey bees until 2010, cell cultures for these pathogens were not available.

In 2011, the German team of Gisder et al., [25] tried to overcome this obstacle and developed a tissue culture model for two obligate pathogens, *N. ceranae* and *N. apis*, based on infection of the heterologous cell line IPL-LD-65Y [64], which was founded from the ovaries of the “gypsy moth” (*Lymantria dispar*).

The results provided a new perspective on the intracellular life cycles of *N. ceranae* and *N. apis*. The finding that obligate bee intracellular pathogens could reproduce in a heterologous cell line is a breakthrough in honey bee pathology and provides a previously unavailable tool to investigate the nature of interactions between honey bees and *Nosema* spp.

The following cell lines were tested for susceptibility to *Nosema* spp. by Gisder et al., 2010 [65] with the positive results: MB-L2 (*Mamestra brassicae*), Schneider-2 (*Drosophila melanogaster*), Sf-9 and Sf-21 (*Spodoptera frugiperda*), SPC-BM-36 (*Bombyx mori*), IPL-LD-65Y (*L. dispar*) and BTI-Tn-5B1–4 (*Trichoplusia ni*). Except for Schneider-2 and HD-AA cells, all cell lines were suitable for *N. ceranae* infection.

However, only the IPL-LD-65Y lepidopteran cell line, which was generated from the ovaries of the *L. dispar* butterfly, could be reproducibly infected by both *N. ceranae* and *N. apis*.

For infectious assays, this cell line was grown in suspension and maintained in TC-100 medium (Invitrogen) supplemented with 11% fetal calf serum (FCS, PromoCell) in tissue culture flasks (Greiner). The cells were incubated at 27 °C in a cooling incubator (Thermo Fisher) and passaged once a week.

Detection of microsporidia in infected cell cultures can take 3 to 10 weeks. Isolation of microsporidia from cell lines as a way to diagnose infection is laborious and time-consuming and tends to fail in case of contaminated samples. Therefore, this diagnosis is performed only by specialized laboratories.

## 9. Prevention and Treatment of Nosematosis

The dependence of the infection on external conditions is as high for *N. apis* as for *N. ceranae*. Therefore, prevention is important to avoid it [11]. For example, hive temperature is a potentially significant factor influencing host resistance to parasites. The presence of nosematosis is generally lower during the summer, since high temperatures in the hive can inhibit the multiplication of *Nosema* spp. [66]. However, the temperature in the hive should reach at least 35 °C, which is rarely reached even right in the center of the hive. Thus, in colder weather, the probability of infection is higher [17].

Another factor supporting the risk of a bee epidemic is hive overcrowding. The infection is even able to disappear on its own at the beginning of the season, when the honey bees are released from hibernation and can defecate outside the hive area.

Equally effective prevention is the appropriate choice of habitat for the overwintering of honey bees, which would allow the bees to fly earlier and more frequently, thus again preventing the hive environment from becoming crowded and at the same time spreading the infection. An economically and especially technically more demanding alternative is artificial flight in a heated room [67].

By exchanging the winter and long-lived generation of honey bees for new, short-lived generations, the beekeeper prevents the premature aging of bees, but it can also help to feed the bee colonies with harmless honey during the production pause in the summer season. Strong hives survive nosematosis better as the burden of the damaged digestive tract with indigestible components of carbohydrate stores is distributed among several bees. The fecal bag does not fill excessively quickly and the honey bee colony lasts until the spring flight without soiling the hive environment [68].

As a precaution, the preparation of replenishment of winter supplies, hygienic feeding and watering, regular disinfection of hives, filtration, incineration of dead individuals, remediation and overall proper care throughout the year are important [69].

After infection, it is necessary to destroy the spores on the mantle and walls of the hive by thermal disinfection. Formidol (formic acid evaporator plates), which is used primarily for the treatment of varrosis, is also available as a means of suppressing nosematosis. Vapors of this acid cause devitalization of spores [70].

## 10. Conclusions

With this work, we wanted to contribute to the knowledge of the pathogenesis and diagnosis of the important honey bee parasite, which is important in view of the alarming global decline in the number of bee hives. Honey bee is of an immense economic and ecological significance, therefore, it is necessary to deal with the factors causing this decline in order to be able to take measures to save honey bees. Nosematosis is the important factor that leads to a weakening of the defenses of hives and, together with other factors, to their collapse.

## Figures and Tables

**Table 1:** Overall incidence and prevalence of *Nosema* spp.

	*Nosema* spp.	*N. apis*	*N. ceranae*	*N. apis* + *N. ceranae* (Mixed Infection)
*N* = 4010	2167	678	1134	356
prevalence	54.0%	31.3%	52.3%	16.4%

**Table 2:** Primers used to diagnose *Nosema* spp. [31].

						Fragment		(bp)
Title	Source		Sequence	Locus	PCR	*N. apis*	*N. bombi*	*N. ceranae*
218MITOC	[12]	fwd rev	CGGCGACGATGTGATATGAAAATATTAA CCCGGTCATTCTCAAACAAAAAACCG	SSU rRNA	qPCR			218–219
321APIS	[12]	fwd rev	GGGGGCATGTCTTTGACGTACTATGTA GGGGGGCGTTTAAAATGTGAAACAACTATG	SSU rRNA	qPCR	321		
BOMBICAR	[1]	fwd rev	GGCCCATGCATGTTTTTGAAGATTATTAT CTACACTTTAACGTAGTTATCTGCGG	SSU rRNA	PCR		101	
ITS	[55]	fwd rev	GATATAAGTCGTAACATGGTTGCT CATCGTTATGGTATCCTATTGATC	ITS region	PCR	120	120	120
N.b.a	[56]	fwd rev	TGCGGCTTAATTTGACTC GGGTAATGACATACAAACAAAC	SSU rRNA/ITS	PCR		511	
Nbombi-SSU-J	[57]	fwd rev	CCATGCATGTTTTTGAAGATTATTAT CATATATTTTTAAAATATGAAACAATAA	SSU rRNA	PCR		323	
NOS	[10]	fwd rev	TGCCGACGATGTGATATGAG CACAGCATCCATTGAAAACG	SSU rRNA	PCR	240		
NosA	[58]	fwd rev	CCGACGATGTGATATGAGATG CACTATTATCATCCTCAGATCATA	SSU rRNA	PCR	209		
SSU-res	[11]	fwd rev	GCCTGACGTAGACGCTATTC GTATTACCGCGGCTGCTGG	SSU rRNA	PCR	402	402	402
NaFor	[59]	Fwd (a)	CTAGTATATTTGAATATTTGTTTACAATGG b	LSU rRNA	qPCR	278		
NcFor		Fwd (c)	TATTGTAGAGAGGTGGGAGATT					316
UnivRev		Urev	GTCGCTATGATCGCTTGCC					
Nosema	[17]	fwd rev	GGCAGTTATGGGAAGTAACA GGTCGTCACATTTCATCTCT	SSU-rRNA	Generic	208		212
*N. ceranae*	[17]	fwd rev	CGGATAAAAGAGTCCGTTACC TGAGCAGGGTTCTAGGGAT	SSU-rRNA	PCR			250
*N. apis*	[17]	fwd rev	CCATTGCCGGATAAGAGAGT CACGCATTGCTGCATCATTGAC	SSU-rRNA	PCR	401		
Nos-16S	[60]	fwd rev	CGTAGACGCTATTCCCTAAGATT CTCCCAACTATACAGTACACCTCATA	SSU-rRNA	PCR	488		488
Mnceranae-F	[31]	fwd	CGTTAAAGTGTAGATAAGATGTT	SSU-rRNA	PCR			
Mnapis-F		fwd	GCATGTCTTTGACGTACTATG					143
Mnbombi-F		fwd	TTTATTTTATGTRYACMGCAG				171	
Muniv-R		Urev	GACTTAGTAGCCGTCTCTC			224		
SSUrRNA-f1b	[61]	Ufwd	CACCAGGTTGATTCTGCCT	SSU-rRNA	Generic		Ca.	
SSUrRNA-r1b		Urev	TGTTCGTCCAGTCAGGGTCGTCA					

ITS: internal transcribed spacer; SSU: rRNA small subunits (SSU), (16S rRNA); N. a.: *Nosema apis*; N. b.: *Nosema bombi*; N. c.: *Nosema ceranae.* (a) Failed to verify fragment size. (b) The sequence was modified to complement the original GenBank entry U97150. Usage: PCR, standard PCR (for detection of various *Nosema* spp.); qPCR, for quantitative or real-time PCR (to quantify different *Nosema* spp.) and standard PCR (to detect different *Nosema* spp.); generic primers amplify all known *Nosema* spp. or all microsporidia without distinction between species.

## References

[1] Brittain C., Williams N., Kremen C., Klein A.M. (2013). Synergistic effects of non-*Apis* bees and honey bees for pollination services. Proc. R. Soc. B Biol. Sci..

[2] Goulson D., Nicholls E., Botías C., Rotheray E.L. (2015). Bee declines driven by combined stress from parasites, pesticides, and lack of flowers. Science.

[3] Tesovnik T., Zorc M., Ristanić M., Glavinić U., Stevanović J., Narat M., Stanimirović Z. (2020). Exposure of honey bee larvae to thiamethoxam and its interaction with *Nosema*
*ceranae* infection in adult honey bees. Environ. Pollut..

[4] Broadrup R.L., Mayack C.H., Schick S.J., Eppley E.J., White H.K., Macherone A. (2019). Honey bee (*Apis mellifera*) exposomes and dysregulated metabolic pathways associated with *Nosema ceranae* infection. PLoS ONE.

[5] Wittner M., Weiss L.M. (1999). The Microsporidia and Microsporidiosis.

[6] Fries I. (1993). *Nosema apis*—A parasite in the honey bee colony. Bee World.

[7] Fries I., Feng F., da Silva A., Slemenda S.B., Pieniazek N.J. (1996). *Nosema ceranae* n. sp. (Microspora, *Nosematidae*), morphological and molecular characterization of a microsporidian parasite of the Asian honey bee *Apis cerana* (Hymenoptera, *Apidae*). Eur. J. Protistol..

[8] Chemurot M., De Smet L., Brunain M., De Rycke R., de Graaf D.C. (2017). *Nosema neumanni* n. sp. (Microsporidia, Nosematidae), a new microsporidian parasite of honeybees, *Apis mellifera* in Uganda. Eur. J. Protistol..

[9] Fries I., Martín-Hernández R., Meana A., GarcíaPalencia P., Higes M. (2006). Natural infections of *Nosema ceranae* in European honey bees. J. Apicultur. Res..

[10] Higes M., Martín R., Meana A. (2006). *Nosema ceranae* a New Microsporidian Parasite in Honeybees in Europe. J. Invertebr. Pathol..

[11] Klee J., Besana A.M., Genersch E., Gisder S., Nanetti A., Tam D.Q., Chinh T.X., Puerta F., Ruz J., Kryger P. (2007). Widespread dispersal of the microsporidian *Nosema ceranae*, an emergent pathogen of the western honey bee, *Apis mellifera*. J. Invertebr. Pathol..

[12] Martin-Hernandez R., Meana A., Prieto L., Salvador A.M., Garrido-Bailon E., Higes M. (2007). Outcome of colonization of apis mellifera by *Nosema ceranae*. Appl. Environ. Microbiol..

[13] Botías C., Anderson D.L., Meana A., Garrido-Bailón E., Martín-Hernández R., Higes M. (2012). Further evidence of an oriental origin for *Nosema ceranae* (Microsporidia: *Nosematidae*). J. Invertebr. Pathol..

[14] Read A.F., Taylor L.H. (2001). The ecology of genetically diverse infections. Science.

[15] Paxton R.J., Klee J., Korpela S., Fries I. (2007). *Nosema ceranae* has infected apis mellifera in europe since at least 1998 and may be more virulent than *Nosema apis*. Apidologie.

[16] Tapaszti Z., Forgách P., Kövágó C., Békési L., Bakonyi T., Rusvai M. (2009). First detection and dominance of *Nosema ceranae* in Hungarian honeybee colonies. Acta Vet. Hung..

[17] Chen Y.P., Evans J.D., Smith J.B., Pettis J.S. (2008). *Nosema ceranae* is a long-present and wide-spread microsporidian infection of the european honey bee (*Apis mellifera*) in the united states. J. Invertebr. Pathol..

[18] Millbrath M.O., van Tran T., Huang W.F., Solter L.F., Tarpy D.R., Lawrence F., Huang Z.Y. (2015). Comparative virulence and competition between *Nosema apis* and *Nosema ceranae* in honey bees (*Apis mellifera*). J. Invertebr. Pathol..

[19] Cox-Foster D.L., Conlan S., Holmes E., Palacios G., Evans J.D., Moran N.A., Quan P.L., Briese T., Hornig M., Geiser D.M. (2007). A metagenomic survey of microbes in honey bee colony collapse disorder. Science.

[20] Zander E., Bottcher F.K. (1984). Krankheiten der Biene.

[21] Bigliardi E., Selmi M.G., Lupetti P., Corona S., Gatti S., Scaglia M., Sacchi I. (1996). Microsporidian spore wall: Ultrastructural findings on *Encephalitozoon hellem* exospore. J. Eukaryot. Microbiol..

[22] Keohane E.M., Weiss L.M., Wittner M., Weiss L.M. (1999). The structure, function, and composition of the microsporidian polar tube. The Microsporidia and Microsporidiosis.

[23] Keeling P.J., Fast N. (2002). Microsporidia: Biology and evolution of highly reduced intracellular parasites. Annu. Rev. Microbiol..

[24] Frixione E., Ruiz L., Santillan M., de Vargas L.V., Tejero J.M., Undeen A.H. (1992). Dynamics of polar filament discharge and sporoplasm expulsion by microsporidian spores. Cell Motil. Cytoskelet..

[25] Gisder S., Möckel N., Linde A., Genersch E. (2011). A cell culture model for *Nosema ceranae* and *Nosema apis* allows new insights into the life cycle of theseimportant honey bee-pathogenic microsporidia. Environ. Microbiol..

[26] Webster T.C. (1993). Nosema apis spore transmission among honey bees. Am. Bee J..

[27] Vidau C., Panek J., Texier C., Biron D.G., Belzunces L.P., Le Gall M., Broussard C., Delbac F., El Alaoui H. (2014). Differential proteomic analysis of midguts from *Nosema ceranae*-infected honeybees reveals manipulation of key host functions. J. Invertebr. Pathol..

[28] Fernandez N., Coineau Y. (2007). Varroa the Serial Bee Killer Mite.

[29] Schmid-Hempel P. (1998). Parasites in Social Insects.

[30] Veselý V., Kubišová S., Haragsim O., Kamler K., Krieg P., Škrobal D., Ptáček V., Titěra D., Peroutková M., Drobníková V. (2003). Beekeeping.

[31] Fries I., Chauzat M.P., Chen Y.P., Doublet V., Genersch E., Gisder S., Higes M., McMahon D.P., Martín-Hernández R., Natsopoulou M. (2013). Standard methods for *Nosema* research. J. Apic. Res..

[32] Fries I., Ekbohm G., Villumstad E. (1984). *Nosema apis*, sampling techniques andhoney yield. J. Apicult. Res..

[33] (2013). Nosemosis of Honey Bees. OIE Terrestrial Manual.

[34] Grozinger C., Robinson G.E. (2015). The power and promise of applying genomics tohoney bee health. Curr. Opin. Insect Sci..

[35] Botías C., Martín-Hernández R., Barrios L., Meana A., Higes M. (2013). *Nosema* spp. Infection and its negative effects on honey bees (*Apis mellifera iberiensis*) at the colonylevel. Vet. Res..

[36] Varis A.L., Ball B.V., Allen M. (1992). The incidence of pathogens in honey bee (*Apismellifera* L.) colonies in Finland and Great Britain. Apidologie.

[37] Gisder S., Schüler V., Horchler L.L., Groth D., Genersch E. (2017). Long-Term Temporal Trends of *Nosema* spp. Infection Prevalence in Northeast Germany: Continuous Spread of *Nosema ceranae*, an Emerging Pathogen of Honey Bees (*Apis mellifera*), but No General Replacement of *Nosema Apis*. Front. Cell. Infect. Microbiol..

[38] Kamler F., Titěra D., Kamler M. (2011). Rozšíření, Patogeneze a Návrh Opatření Vchovech Včel Ohrožených Mikrosporidií Nosema Ceranae.

[39] Staroň M., Jurovčíková J., Čermáková T., Staroňová D. (2012). Scientific note on incidence of Nosema apis and Nosema ceranae in Slovakia during the years 2009 and 2010. Slovak J. Anim. Sci..

[40] Csáki T., Heltai M., Markolt F., Kovács B., Békési L., Ladányi M., Péntek-Zakar E., Meana A., Botías C., Martín-Henández R. (2015). Permanent prevalence of *Nosema ceranae* in honey bees (*Apis mellifera*) in Hungary. Acta Vet. Hung..

[41] Papini R., Mancianti F., Canovai R., Cosci F., Rocchigiani G., Benelli G., Canale A. (2017). Prevalence of the microsporidian *Nosema ceranae* in honeybee (*Apis mellifera*) apiaries in Central Italy Saudi. J. Biol. Sci..

[42] Shumkova R., Georgieva A., Radoslavov G., Sirakova D., Dzhebir G., Neov B., Bouga M., Hristov P. (2018). The first report of the prevalence of *Nosema ceranae* in Bulgaria. PeerJ.

[43] Matthijs S., De Waele V., Vandenberge V., Verhoeven B., Evers J., Brunain M., Saegerman C., De Winter P.J.J., Roels S., de Graaf D.C. (2020). Nationwide Screening for Bee Viruses and Parasites in Belgian Honey Bees. Viruses.

[44] Porrini C., Mutinelli F., Bortolotti L., Granato A. (2016). The Status of Honey Bee Health in Italy: Results from the Nationwide Bee Monitoring Network. PLoS ONE.

[45] van Gool T., Snijders F., Reiss P., Eeftinck Schattenkerk J.K., van den Bergh Weerman M.A., Bartelsman J.F., Bruins J.J., Canning E.U., Dankert J. (1993). Diagnosis of intestinal and disseminated microsporidial infections in patients with HIV by a new rapid fluorescence technique. J. Clin. Pathol..

[46] (2018). Chapter 3.02.04 Nosemosis 2018. Terrestrial Manual of the OIE.

[47] Weber R., Bryan R.T., Owen R.L., Wilcox C.M., Gorelkin L., Visvesvara G.S. (1992). Improved light-microscopical detection of microsporidia spores in stool and duodenal aspirates. The Enteric Opportunistic Infections Working Group. N. Engl. J. Med..

[48] Vávra J., Dahbiová R., Hollister W.S., Canning E.U. (1993). Staining of microsporidian spores by optical brighteners with remarks on the use of brighteners for the diagnosis of AIDS associated human microsporidioses. Folia Parasitol..

[49] Chen Y.P., Huang Z.Y. (2010). *Nosema ceranae*, a newly identified pathogen of *Apis mellifera* in the USA and Asia. Apidologie.

[50] Bokaie S., Sharifi L., Mehrabadi M. (2014). Prevalence and epizootical aspects of varroasis in Golestan province, northern Iran. J. Arthropod-Borne Dis..

[51] Ryan N.J., Sutherland G., Coughlan K., Globan M., Doultree J., Marshall J., Baird R.W., Pedersen J., Dwyer B. (1993). A new trichrome-blue stain for detection of microsporidial species in urine, stool, and nasopharyngeal specimens. J. Clin. Microbiol..

[52] Fedorko D.P., Hijazi Y.M. (1996). Application of molecular techniques to the diagnosis of microsporidial infection. Emerg. Infect. Dis..

[53] Burges H.D., Canning E.U., Hulls I.K. (1974). Ultrastructure of *Nosema oryzaephili* and the taxonomic value of the polar filament. J. Invertebr. Pathol..

[54] Evans J.D., Schwarz R.S., Chen Y.P., Budge G., Cornman R.S., Rua P.D., la Miranda J.R., de Foret S., Foster L., Gauthier L. (2013). Standard methods for molecular research in *Apis mellifera*. J. Agricult. Res..

[55] Plischuk S., Martín-Hernández R., Prieto L., Lucía M., Botías C., Meana A., Abrahamovich A.H., Lange C., Higes M. (2009). South American native bumblebees (Hymenoptera: Apidae) infected by *Nosema ceranae* (*Microsporidia*), an emerging pathogen of honeybees (*Apis mellifera*). Environ. Microbiol. Rep..

[56] Klee J., Tay W.T., Paxton R.J. (2006). Specific and sensitive detection of *Nosema bombi* (Microsporidia: Nosematidae) in bumble bees (Bombus spp.; Hymenoptera: Apidae) by PCR of partial rRNA gene sequences. J. Invertebr. Pathol..

[57] Erler S., Lommatzsch S., Lattorff H.M.G. (2012). Comparative analysis of detection limits and specificity of molecular diagnostic markers for three pathogens (*Microsporidia*, *Nosema* spp.) in the key pollinators *Apis mellifera* and *Bombus Terrestris*. Parasitol. Res..

[58] Webster T.C., Pomper K.W., Hunt G., Thacker E.M., Jones S.C. (2004). *Nosema apis* infection in worker and queen *Apis mellifera*. Apidologie.

[59] Forsgren E., Fries I. (2010). Comparative Virulence of Nosema Ceranae and Nosema Apis in Individual European Honey Bees. Vet. Parasitol..

[60] Stevanovic J., Stanimirovic Z., Genersch E., Kovacevic S.R., Ljubenkovic J., Radavic M., Aleksic N. (2011). Dominance of *Nosema ceranae* in honey bees in the Balkan countries in the absence of symptoms of colony collapse disorder. Apidologie.

[61] Tay W.T., O’Mahoney E.M., Paxton R.J. (2005). Complete rRNA gene sequences reveal that the microsporidium *Nosema bombi* infects diverse bumble bee (*Bombus* spp.) hosts and contains multiple polymorphic sites. J. Eukaryot. Microbiol..

[62] Chen Y., Evans J.D., Zhou L., Boncristiani H., Kimura K., Xiao T., Litkowski A.M., Pettis J.S. (2009). Asymmetrical coexistence of *Nosema ceranae* and *Nosema apis* in honey bees. J. Invertebr. Pathol..

[63] Carreck N.L., Ratnieks F.L.W. (2013). Will neonicotinoid moratorium save the bees?. Res. Fortn..

[64] Goodwin R.H., Tompkins G.J., McCawley P. (1978). Gypsy moth cell lines divergent in viral susceptibility. I. Culture and identification. Vitro.

[65] Gisder S., Hedtke K., Möckel N., Frielitz M.C., Linde A., Genersch E. (2010). Five-year cohort study of *Nosema* spp. in Germany: Does climate shape virulence and assertiveness of *Nosema ceranae*?. Appl. Enviro. Microbiol..

[66] Holt H.L., Aronstein K.A., Grozinger C.M. (2013). Chronic parasitization by *Nosema* microsporidia causes global expression changes in core nutritional, metabolic andbehavioral pathways in honey bee workers (*Apis mellifera*). BMC Genom..

[67] Schmid-Hempel R., Tognazzo M. (2010). Molecular divergence defines two distinctlineages of *Crithidia bombi* (*Trypanosomatidae*), parasites of bumblebees. J. Eukaryot. Microbiol..

[68] Bradbear N. (2009). Bees and Their Role in Forest Livelihoods.

[69] Muñoz I., Cepero A., Pinto M.A., Martín-Hernández R., Higes M., De la Rúa P. (2014). Presence of *Nosema ceranae* associated with honeybee queen introductions. Infect. Genet. Evol..

[70] Evans J.D., Aronstein K., Chen Y.P., Hetru C., Imler J.L., Jiang H., Kanost M., Thompson G.J., Zou Z., Hultmark D. (2006). Immune pathways and defence mechanisms in honeybees *Apis mellifera*. Insect Mol. Biol..

